# Efficacy of a novel endoscopically deliverable muco-adhesive hemostatic powder in an acute gastric bleeding porcine model

**DOI:** 10.1371/journal.pone.0216829

**Published:** 2019-06-11

**Authors:** ByoungWook Bang, Eunhye Lee, JinHee Maeng, Keunsu Kim, Joo Ha Hwang, Suong-Hyu Hyon, Woogi Hyon, Don Haeng Lee

**Affiliations:** 1 Division of Gastroenterology, Department of Internal Medicine, Inha University College of Medicine, Incheon, Republic of Korea; 2 Utah-Inha DDS and Advanced Therapeutics Research Center, Incheon, Republic of Korea; 3 Division of Gastroenterology and Hepatology, Stanford University School of Medicine, Palo Alto, California, United States of America; 4 BMG Incorporated, Kyoto, Japan; 5 School of Materials Science, Japan Advanced Institute of Science and Technology, Nomi, Ishikawa, Japan; Chung-Ang University College of Engineering, REPUBLIC OF KOREA

## Abstract

This study investigated the effectiveness of new hemostatic adhesive powder (UI-EWD) in a swine mode of acute gastric bleeding. Gastric ulcer bleeding was induced endoscopically at two locations in each of eight heparinized mini-pigs. UI-EWD and saline were sprayed endoscopically in the experimental (n = 5) and control groups (n = 3), respectively. The hemostatic effect and hydrogel persistence on ulcers were periodically evaluated endoscopically. Initial hemostasis was achieved successfully in all lesions in the experimental group. Follow-up endoscopy showed minor delayed bleeding in 10% at 6 hours in the experimental group, whereas re-bleeding was observed in 50% at 6 hours in the control group. UI-EWD gel persisted at 90%, 80%, and 50% of ulcer bases at 6, 18, and 42 hours post-application, respectively. This study suggests that muco-adhesive UI-EWD may be effective in the endoscopic treatment of active ulcer bleeding.

## Introduction

Gastrointestinal (GI) bleeding is a common condition that leads to hospital admission, and significant morbidity and mortality [1]. Endoscopic hemostasis is accepted as the first-line treatment for GI bleeding. Various endoscopic hemostatic methods such as epinephrine injection, thermal coagulation, and mechanical hemostasis are currently used as single or combination therapies [2]. Although they are highly effective in achieving initial hemostasis (up to 85–95%) [3], the endoscopic management of GI bleeding is often challenging as it depends on lesion location and on the extent and characteristics of bleeding. Accordingly, in such cases, a high level of technical expertise is often required. However, life-threatening GI bleeding can occur at any time, and less-skilled endoscopists may have difficulty achieving endoscopic hemostasis. Therefore, despite major advances in endoscopic hemostatic methods, simple and effective methods are still needed.

Recently, hemostatic powders have been introduced to treat upper GI bleeding. Currently, three hemostatic agents are commercially available for endoscopic purposes [4], that is, TC-325 (Hemospray, Cook Medical, Winston-Salem, NC, USA) [5, 6], Endoclot (EndoClot Plus, Inc, Santa Clara, CA, USA) [7] and Ankaferd Blood stopper (Ankaferd Health Products Ltd., Turkey). These products have different characteristics and mechanisms of action that stem from their chemical compositions. Nevertheless, when they come into contact with liquid, they immediately convert from a powder into a gel and form a stable mechanical barrier that can seal bleeding foci and enhance clot formation, inducing hemostasis^4^. A large multicenter registry trial of TC-325 revealed an initial hemostasis rate of 96.5% and a re-bleeding rate over the first postoperative week of 26.7%^6^. Ankaferd Blood stopper [8] and Endoclot [9] have been reported to have initial hemostasis rates of 83.3% and 64%, respectively. However, despite the excellent outcomes reported by several clinical studies, these products have several drawbacks. In particular, the gels formed have insufficient adhesion to maintain hemostasis and dissolve too rapidly. As a result, their hemostatic effect last for only up to one day, and re-bleeding rates are high [10]. In addition, deployment of the powder often is complicated by premature gel formation resulting in clogging of the catheter and indiscriminate dispersion of the powder from the tip of the catheter obscuring the field of vision.

We developed an endoscopically applicable hemostatic adhesive powder (UI-EWD, Next Biomedical, Incheon, Korea). UI-EWD consists of oxidized dextran and succinic acid modified amino acid, and is thus, biodegradable and biocompatible. When UI-EWD contacts water, the aldehyde group of the oxidized dextran reacts with the amine of the succinic acid modified amino acid, and becomes an adhesive gel. The gel physically adheres to the ulcer base creating a mechanical barrier to achieve hemostasis.

The aims of this study were to investigate 1) the hemostatic ability of UI-EWD and 2) its persistence after gel formation on ulcer bases in a porcine model of acute gastric bleeding.

## Materials & methods

### Material

**Fig 1:**
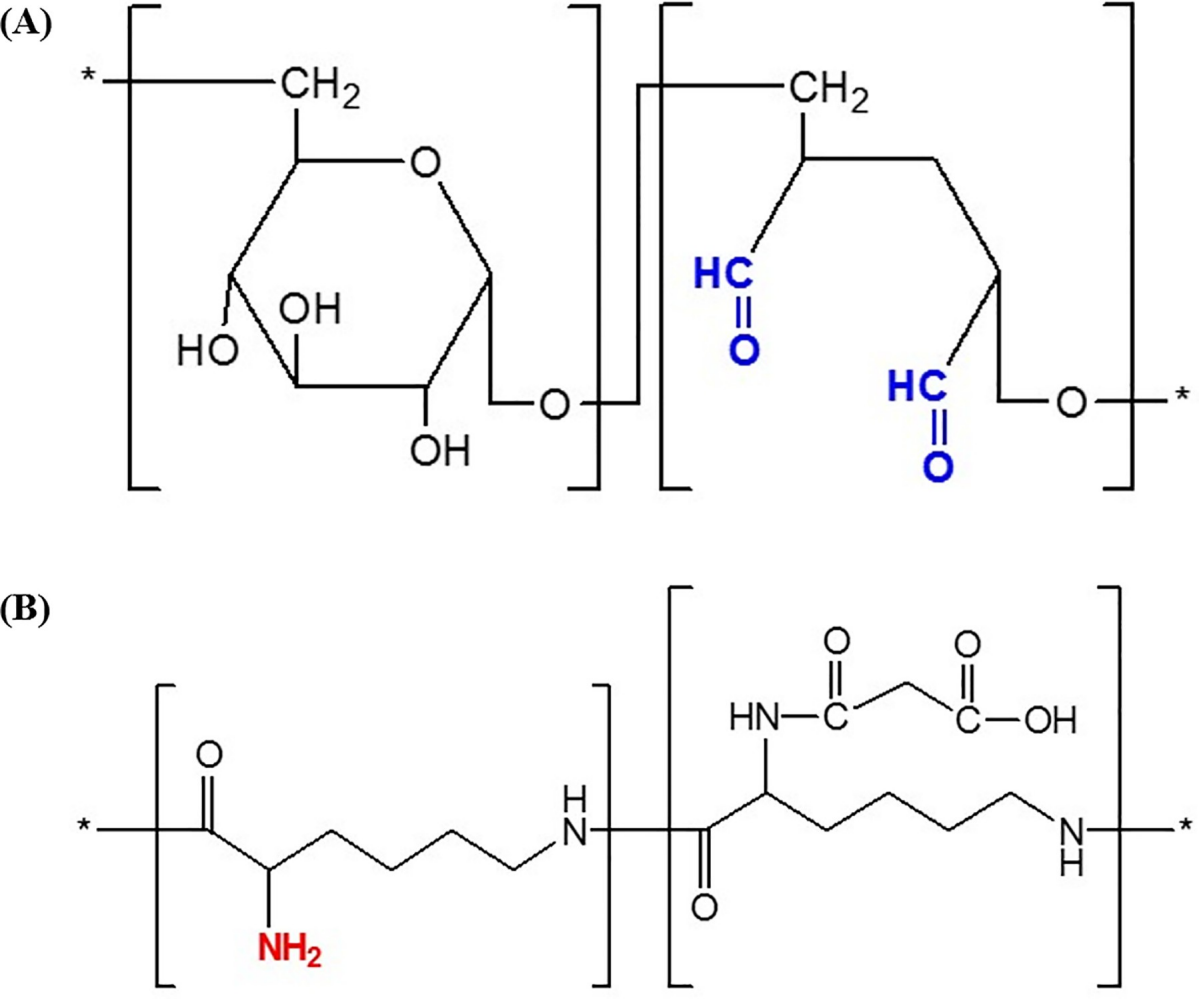
Composition of UI-EWD. (A) Chemical structures of oxidized dextran and (B) succinic acid modified amino acid.

UI-EWD is a biocompatible natural polymer that consists of oxidized dextran and succinic acid modified amino acid (Fig 1) [11–14]. These two materials, when brought into contact with water, are immediately converted to a highly adhesive gel. Hydrogels were easily formed by the reaction between the aldehyde (oxidized dextran) and amino (ε-poly-L-lysine) groups, leading to the formation of a Schiff base and multiple crosslinking points, and these hydrogels showed high adhesive strength. These two components lead to the formation of cohesion bonds inherent within the hydrogel by Schiff base reaction, and adhesion bonds between the hydrogel and the tissue by the same chemical reaction.

**Fig 2:**
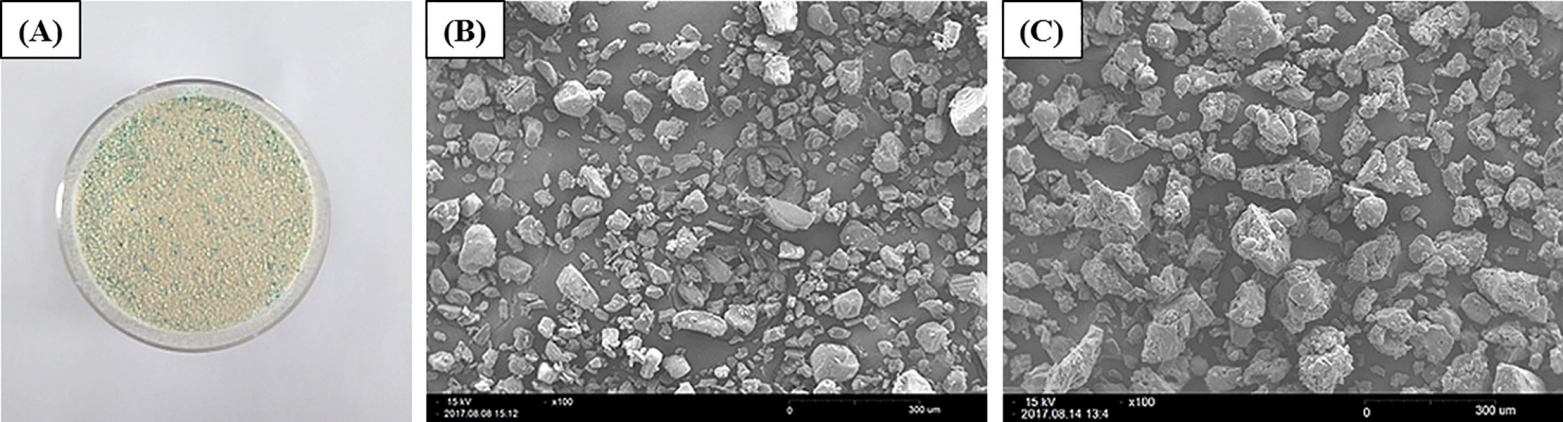
Images of UI-EWD granules. (A) Macroscopic image of coated UI-EWD. (B) Microscopic image of uncoated UI-EWD and (C) coated UI-EWD after the coating process by scanning electron microscopes Scale bars, 300 μm.

However, unmodified UI-EWD (Fig 2) is difficult to deliver endoscopically due to its highwater absorption characteristic. Thus, we modified the water absorption capacity of unmodified UI-EWD using coating technology (Fig 2). The coating process was conducted by using a fluidized bed granulator with the following liquid coating materials: Eudragit (EVONIK, Essen, Germany), magnesium stearate, and butylated hydroxy anisole (Sigma-Aldrich, Saint Lousis, MO, USA).

### Physical properties of UI-EWD gel

The adhesion force of UI-EWD gel was measured using a texture analyzer (TXA, Yeonjin Corporation, Seoul, South Korea) under the following conditions: pre-load 10 gf, descent rate 0.5 mm/sec, and maximum load 600 gf in triplicate. Gelation of UI-EWD was induced by adding 250 μl of water to 50 mg of powder. The water absorption capacity of UI-EWD was previously evaluated using ASTM D570 [15]. Briefly, 40 ml water was added to 1g of UI-EWD and the resulting gel was incubated at 37°C for 30 min. Surplus water was then removed and water absorption (%) was calculated using the following equation;

WaterAbsorption(%)=[(Wetweight‐Dryweight)/Dryweight]x100

### Animals

For the acute hemostasis study, eight male mini-pigs weighing 35 kg to 40 kg were used. This study was carried out in strict accordance with the recommendations in the Guide for the Care and Use of Laboratory Animals of the National Institutes of Health. The protocol was approved by the Committee on the Ethics of Animal Experiments of the Inha University (Protocol Number: INHA-130822-234). All procedure was performed under tiletamine/zolazepam/ xylazine anesthesia and isoflurane inhalation anesthesia, and all efforts were made to minimize suffering. All animals were fasted overnight but allowed free access to water for 24 hours before the endoscopic procedure. Pre-anesthesia procedures conducted by an intramuscular injection of 0.1 ml/kg of the 1:1 mixture of Xylazine hydrochloride (Rompun, Bayer, Germany)/zolazepam (Zoletil 50, Virbac, France). The general anesthesia was achieved by 5–10 ml/kg/min of the 1.5% to 2.0% isoflurane (Ifran Hana Pharm Co Ltd, South Korea) inhalation delivered by endotracheal intubation and mechanical ventilation.

#### Induction of endoscopic submucosal dissection-induced bleeding gastric ulcers in mini-pigs

Heparin (200 IU/kg) was intravenously injected before the endoscopic procedure. With the animal in the left lateral decubitus position, an endoscope (GIF-Q260; Olympus Medical Systems, Tokyo, Japan) was advanced into the stomach. Two gastric ulcers were produced per animal. Target areas were marked with an argon plasma coagulator, and isotonic saline was injected into the submucosal layer. Endoscopic submucosal dissection (ESD) was performed to create gastric ulcers ~2 cm in diameter. To maximize bleeding, hemostasis was not performed during ESD.

#### Evaluation of hemostatic efficacy and gel persistence at ulcer bases in mini-pigs

Three animals were assigned to the control group and five to the test group. Normal saline was sprayed on ESD sites in the control group. In the test group, three to six grams of UI-EWD was applied to ESD-induced ulcers via a delivery catheter using the Alto shooter (Kaigen Co., Ltd, Osaka, Japan). After the procedure, animals were allowed to recover in individual cages, but were not fed for 24 h. No other treatments were administered, such as proton pump inhibitors or antibiotics, during the entire study. Follow-up endoscopy was performed at 6, 18, 42 and 66 hours after procedure to evaluate re-bleeding rates and gel persistence on ESD sites. At the end of the observation period, animals were euthanized by injecting Succinylcholine and necropsy was performed.

#### Statistical analysis

The primary end point of this study was the proportion of pigs in which hemostasis had been achieved at 10 min for initial bleeding rate. Re-bleeding rates and gel persistence rates were also compared between the two groups. Fisher’s exact for continuous variables was applied. A *P* value of less than 0.05 was considered statistically significant. Sample size calculations were based the results of a prior pilot study in gastric bleeding pig model in our laboratory (unpublished data). In our initial experiments, the initial hemostatic rate was 100% (n = 3) in the treatment group versus 0% in the control group. From this initial experimental data, it was calculated that a sample size of more than 5 in each group was required to detect a difference between the two groups.

All calculations were done using the GraphPad Prism 6.0 statistical software (GraphPad software, San Diego, CA, USA).

## Results

### Physical properties of UI-EWD

When the adhesive force of UI-EWD gel was compared with those of existing commercial products, the average adhesive force of UI-EWD was 53.7 gf while those of Hemospray and Endoclot were 26.9 gf and 19.1 gf respectively. Gel strength and water absorption capacity of UI-EWD were 0.04962 ± 0.0001 MPa and 409 ± 16%, respectively.

### The initial and permanent hemostatic effects of UI-EWD in the mini-pig model

**Fig 3:**
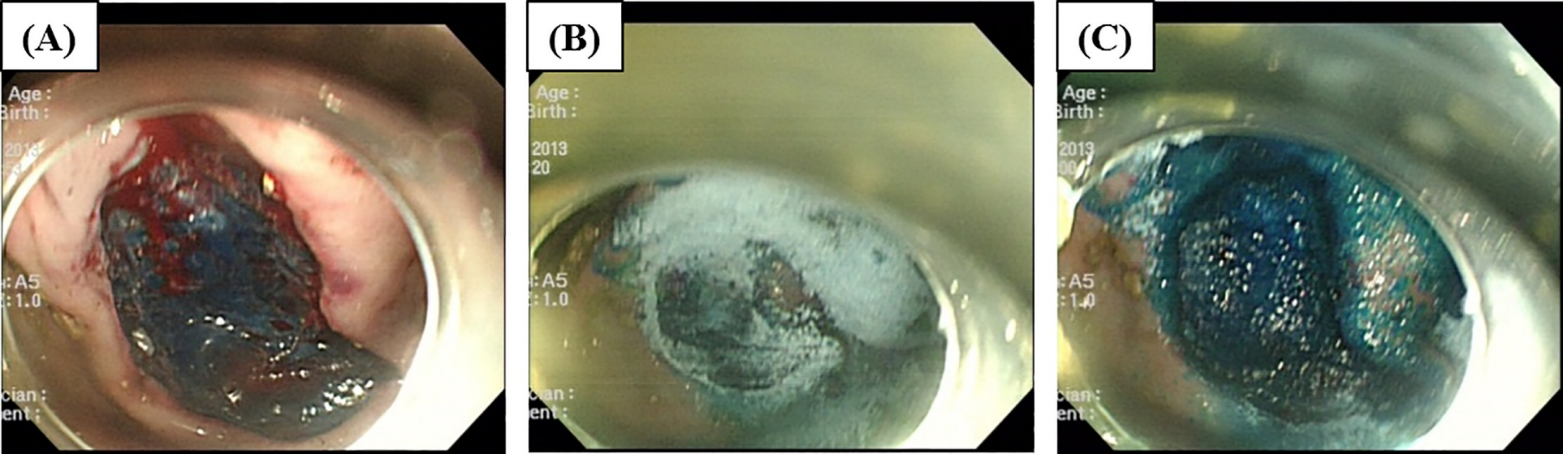
The representative endoscopic images of UI-EWD application after endoscopic submucosal dissection (ESD). (A) The animal has a Forrest Ib bleeding after ESD. (B), (C) The application of UI-EWD at the bleeding site. (D) After application of the powder, hydrogel was firmly attached at the bleeding site without any further sign of bleeding.

**Fig 4:**
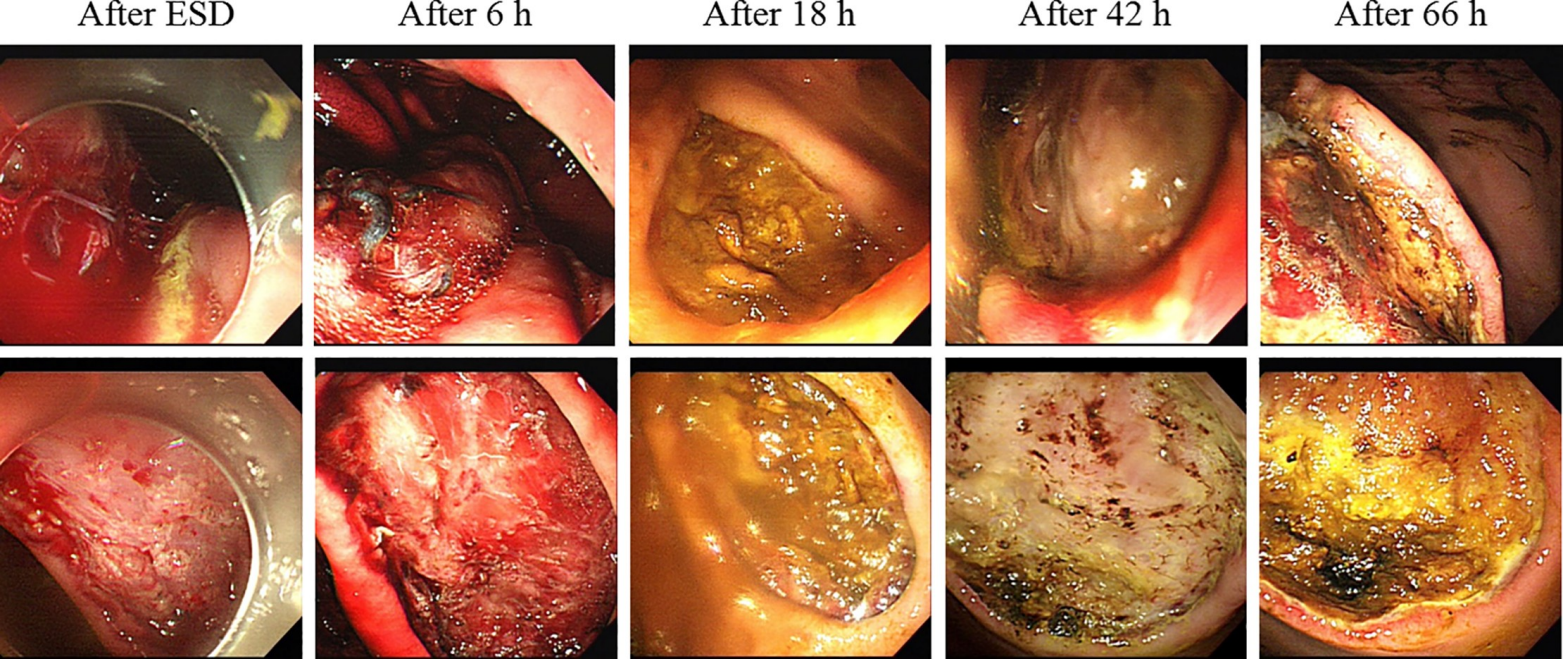
The endoscopic images of control group during 66 hours follow-up endoscopy after ESD. Bleeding (Forrest Ib) was observed in ESD sites at 6, 42, and 66 hours.

**Fig 5:**
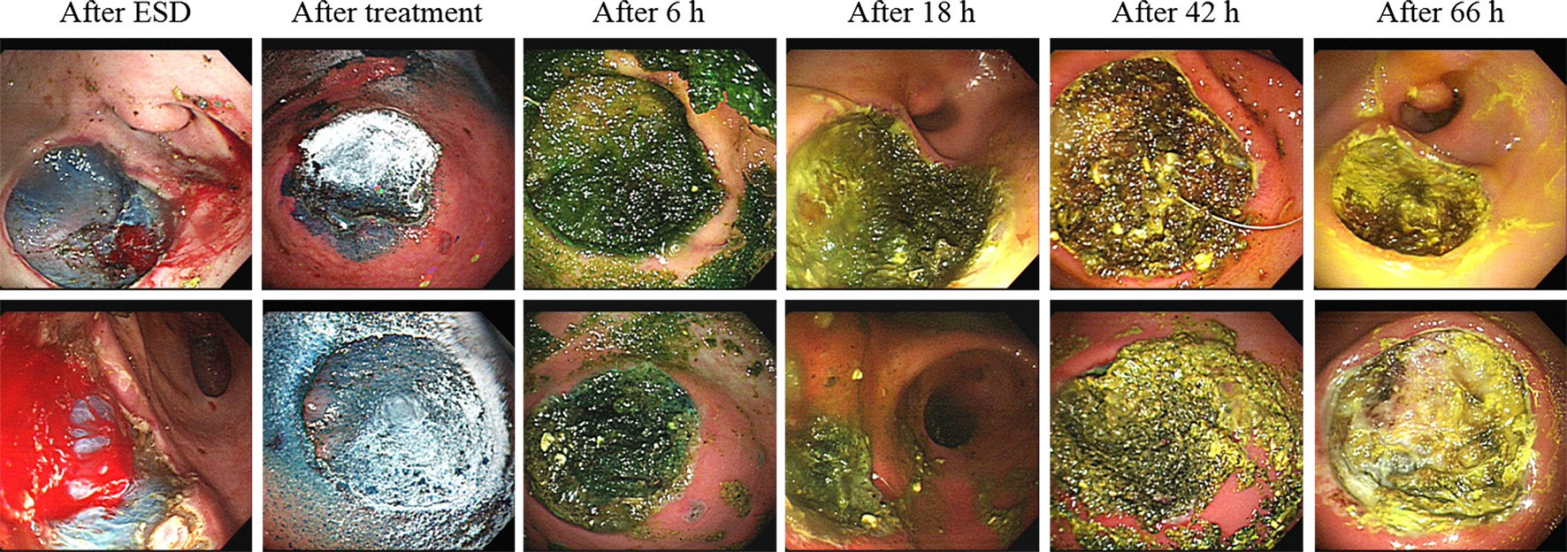
Adhesion of UI-EWD gel to ulcers over time. UI-EWD gel was attached to ulcers at 18 hours and 42 hours after spraying, but not observed at 66 hours after spraying.

No procedure-related adverse events were observed in any pig. Bleeding was induced in all ulcers due to heparinization before ESD. UI-EWD was sprayed onto active bleeding sites using an Alto shooter, which immediately induced hemostasis at all ESD lesions (Fig 3 and Table 1). The initial hemostatic rate in the treated group was 100% compared to the Non-treated (control) group with 0% (*P* <0.001). ESD sites were observed endoscopically at 6, 18, 42, and 66 hours after treatment. In the control group, persistent bleeding (Forrest Ib) was observed in 50% of ESD sites (3/6) at 6 hours after ESD. There was no evidence of bleeding in the control group at 18 hours; however, there was evidence of re-bleeding at one site at 42 and 66 hours after ESD in the control group (Fig 4). In the UI-EWD group, bleeding (Forrest Ib) was observed in one lesion at 6 h after ESD, which was believed to have been a result of early loss of adhesion of the gel to the ESD site. However, no further bleeding was observed at 18, 42 or 66 hours after ESD in the group treated with UI-EWD (Fig 5, Table 1, and S1 Table). This result showed no significant difference from the control group.

**Table 1:** Bleeding rates in ESD-induced gastric ulcers.

Bleeding Type	Time (hour)	Control (bleeding number/total N number)	UI-EWD (bleeding number/total N number)
Initial bleeding	0	100% (6/6)	0% *** (0/10)
Re-bleeding	6	50% (3/6)	10% (1^#^/10)
	18	0% (0/6)	0% (0/10)
	42	17% (1/6)	0% (0/10)
	66	17% (1/6)	0% (0/10)

^#^ Bleeding was caused by early detachment of the UI-EWD gel from an ulcer.

*** *P* <0.001 compare to Control group.

### Gel persistence

In terms of gel persistence, the UI-EWD gel remained attached to 90% (*P* <0.001 compared with the control group) of lesions at 6 hours, 80% (*P* <0.01 compared with the control group) at 18 hours, 50% at 42 hours, and was completely dislodged by 66 hours after ESD (Fig 5, Table 2, S2 Table). In these results, gel persistence rate at 6 hours and 18 hours were significant differences compare with the control group (*P* <0.001 and *P* <0.01, respectively).

**Table 2:** Persistence rates of UI-EWD gel on ESD-induced gastric ulcers.

Time (hour)	0	6	18	42	66
UI-EWD (site = 10)	100%***	90%*** (9/10)	80%** (8/10)	50% (5/10)	0%

*** *P* <0.001 and

** *P* <0.01 compare to Control group.

## Discussion

Hemostatic powders are promising tools for endoscopic treatment of GI bleeding with the potential benefits of precise targeting, ability to treat areas that are large or in difficult location, and ease of use. To our knowledge, three endoscopically deliverable hemostatic powders are currently available: TC-325 (Hemospray, Cook Medical) is a proprietary, inorganic, absorbent powder; Ankaferd Blood Stopper (Ankaferd Health Products Ltd, Istanbul, Turkey) [16] is composed of a traditional Turkish herbal mixture; and EndoClot (EndoClot Plus, Inc) [17] is derived from purified plant starch. In terms of hemostaic mechanisms, EndoClot and TC-325 rapidly absorb water from blood, and act cohesively and adhesively to form a mechanical plug over bleeding sites and accelerate the clotting cascade. Ankaferd Blood Stopper rapidly forms an encapsulated protein network with vital erythroid aggregation^4^. In addition, several types of hemostatic powder based on chitosan and epidermal growth factor have been reported in the literature [18, 19].

Although initial reports on the use of hemostatic powder sprays to manage GI bleeding are promising [5, 8, 9], their efficacy may be limited for several reasons. Although several clinical studies have reported high initial hemostasis rates, the adhesive strengths of commercial hemostatic powders are considered to be relatively low and inadequate for durable hemostasis. Furthermore, delayed bleeding is likely to occur because hemostatic powders do not induce healing of bleeding foci and appear to be lost as early as 12 hours after application. The rebleeding rate after TC-325 spray application has been reported to range from 10% to 38.9% [5, 6, 20], and thus, its use requires second-look endoscopy. With some powders it is difficult to target the delivery due to excessive over-spraying when delivered by a catheter. Although precise targeting is unnecessary, hemostatic powder deposition can interfere with endoscopic visualization. Lastly, certain formulations of powders have issues with frequent blockage of delivery catheters. UI-EWD addresses these existing problems of hemostatic powders in several ways. It is more adhesive than commercially available products, and therefore, is expected to achieve higher durable hemostasis rates. UI-EWD hydrogel persists on most ulcers for more than 18 hours as demonstrated in this study with attachment rates of 80% at 18 hours and 50% at 42 hours. UI-EWD has a coating to decrease the initial rate of water absorption to prevent blockage of the delivery catheter from premature gel formation and to optimize the particle size to enhance the spraying characteristics resulting precise targeting of lesions. However, like other hemostatic powders, the endoscopic working channel must be flushed with air before introducing the catheter and direct contact of the catheter tip with any fluid must be avoided [21].

Commercial hemostatic powders are inorganic powders that depend on clot formation to achieve hemostasis [21], and thus, they do not attach to non-bleeding surfaces and only affect areas of active bleeding. UI-EWD does not require active bleeding for adhesion because its adhesive characteristics are a result of the reversible cross-linking of amine and aldehyde groups when they come into contact with moisture. For this reason, UI-EWD can be used to treat non-bleeding visible vessels and prevent delayed bleeding after primary hemostasis has been achieved after treatment of ulcer bleeding or after ESD or EMR.

In terms of safety, UI-EWD contains no human or animal derived components. It is biologically inert, non-toxic, and does not appear to interact with tissues. Furthermore, it is not absorbed by the body and it exits via the small intestine within 66 hours. Although we gave 1,000 mg/kg of UI-EWD orally to beagle dogs, no adverse clinical symptoms or biochemical changes were observed (data not shown). All items were tested for toxicity in accordance with ISO-10993, the universal standard for the safety of medical devices, and as a result, safety was confirmed.

Several limitations of this study bear consideration. First, it was undertaken on animals, and thus, clinical trials are needed to demonstrate the effectiveness of UI-EWD in humans. Second, ESD-induced bleeding stopped spontaneously in most cases in our anticoagulated swine model regardless of UI-EWD application. Although heparin was administered to induce severe bleeding, severe bleeding and hemorrhage-related events were not observed. Despite the differences between our porcine animal model and humans, we did observe a higher hemostasis rate in the UI-EWD treated experimental group than in the control group.

## Conclusions

In conclusion, this study shows that UI-EWD is effective for hemostasis due to its adhesive ability and prolonged duration of adhesion on ulcers.

## Supporting information

S1 Table Raw data of UI-EWD bleeding rates. (PDF)

S2 Table Raw date of UI-EWD gel persistence rates. (PDF)
