# Supplementary material for: Efficacy of a novel endoscopically deliverable muco-adhesive hemostatic powder in an acute gastric bleeding porcine model
Source: PLoS One. 2019 Jun 11;14(6):e0216829. doi: 10.1371/journal.pone.0216829 (PMC6559629; doi:10.1371/journal.pone.0216829)
Supplement: S1 Table — (PDF) [file pone.0216829.s001.pdf]

	Group / Time (hour)	Control			UI-EWD		
		Bleeding number	Total number	Bleeding rate (%)	Bleeding number	Total number	Bleeding rate (%)
Initial bleeding	0	6	6	100	0	10	0
Re-bleeding	6	3	6	50	1	10	10
	18	0	6	0	0	10	0
	42	1	6	17	0	10	0
	66	1	6	17	0	10	0
