# Supplementary material for: Efficacy of a novel endoscopically deliverable muco-adhesive hemostatic powder in an acute gastric bleeding porcine model
Source: PLoS One. 2019 Jun 11;14(6):e0216829. doi: 10.1371/journal.pone.0216829 (PMC6559629; doi:10.1371/journal.pone.0216829)
Supplement: S2 Table — (PDF) [file pone.0216829.s002.pdf]

Group	No.	OH	6H	18H	42H	66H
Control	1	X	X	X	X	X
	2	X	X	X	X	X
	3	X	X	X	X	X
	4	X	X	X	X	X
	5	X	X	X	X	X
	6	X	X	X	X	X
UI-EWD	1	O	O	O	O	X
	2	O	X	X	X	X
	3	O	O	X	X	X
	4	O	O	O	X	X
	5	O	O	O	O	X
	6	O	O	O	X	X
	7	O	O	O	X	X
	8	O	O	O	O	X
	9	O	O	O	O	X
	10	O	O	O	O	X
